# Challenges and New Directions in Therapeutic Cancer Vaccine Development

**DOI:** 10.3390/vaccines12121341

**Published:** 2024-11-28

**Authors:** Danjie Pan, Jiayang Liu, Xuan Huang, Songna Wang, Kudelaidi Kuerban, Yan Yan, Yi Zhun Zhu, Li Ye

**Affiliations:** 1 Laboratory of Drug Discovery from Natural Resources and Industrialization, School of Pharmacy, Macau University of Science and Technology, Macau 999078, China; 21211030089@m.fudan.edu.cn (D.P.);; 2 Department of Biological Medicines, School of Pharmacy, Fudan University, Shanghai 201203, China; 22111510062@m.fudan.edu.cn (J.L.);

**Keywords:** cancer vaccine, anonymous antigen vaccines, predefined antigen vaccine, tumor immunotherapy, immunotherapy drug

## Abstract

Tumor vaccine is a promising immunotherapy for solid tumors. Therapeutic tumor vaccines aim at inducing tumor regression, establishing durable antitumor memory, and avoiding non-specific or adverse reactions. However, tumor-induced immune suppression and immune resistance pose challenges to achieving this goal. In this article, we review multiple challenges currently faced in the development of therapeutic tumor vaccines, with a particular focus on anonymous antigen vaccines in situ as a new direction. We summarize the research progress in this area, aiming to provide a reference for future studies on tumor vaccines.

## 1. Introduction

Tumor immunotherapy refers to re-activating the immune system of patients to fight against tumors, in which immune cells play critical roles [1]. For an effective antitumor immune response to eliminate cancer cells, a series of sequential steps termed the “cancer- immunity cycle” must be initiated [2]. Within this cycle, antigen-presenting cells (APCs) first process antigens derived from tumor cells. Subsequently, dendritic cells (DCs) present these antigens on major histocompatibility complex (MHC) class I and II molecules to T cells, thereby initiating and activating effector T cells against tumor-specific antigens. Ultimately, activated effector T cells recognize and attack tumor cells through their T-cell receptors (TCRs) [2,3,4]. Several tumor immunotherapies have entered clinical application, such as immune checkpoint blockade (ICB) and chimeric antigen receptor T-cell (CAR-T) therapy, each targeting different stages of the cancer-immunity cycle [5,6]. However, the efficacy of first-generation immune checkpoint inhibitors (ICIs), like PD-1/PD-L1 antibodies, is limited by the infiltration of cytotoxic T lymphocytes (CTLs) into tumors [7,8]. Additionally, the clinical application of CAR-T therapy is restricted by high costs, time requirements, and adverse reactions [9]. The presence of myeloid-derived suppressor cells (MDSCs), tumor-associated macrophages (TAMs), and regulatory T cells (Tregs) at tumor sites would also lead to poor treatment outcomes. While these methods target the latter stages of the cancer-mmunity cycle, tumor vaccines focus on the upstream of this cycle by inducing CTLs and humoral immune responses through tumor antigens, and are therefore regarded as promising candidates for activating the immune system [10,11].

**Figure 1:**
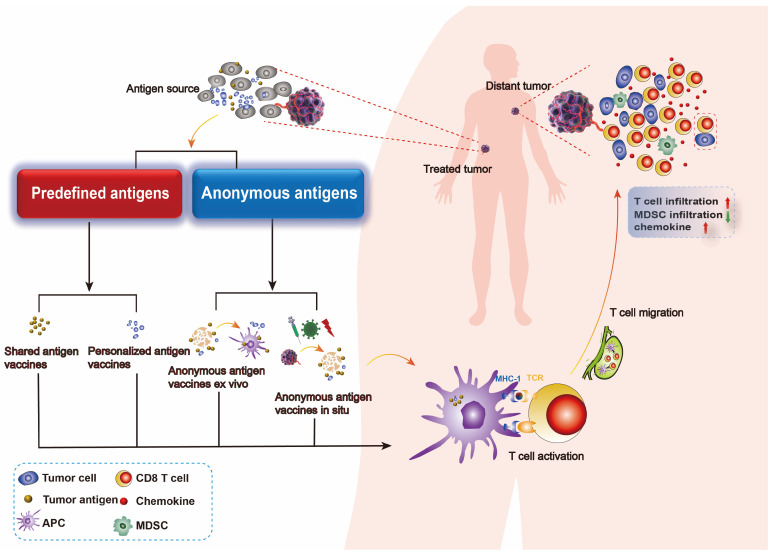
Types of tumor vaccine. Tumor vaccines are prepared from tumor antigen, thereby they could activate specific antitumor immune responses, including an increase in chemokine expression and T-cell infiltration within the tumor and a decrease in MDSC infiltration. Different sources of tumor antigens draw a distinction between predefined antigen vaccines and anonymous antigen vaccines. Predefined antigen vaccines consist of shared antigen vaccines and personalized antigen vaccines, both of which require the preparation of a certain tumor antigen for the vaccine. Anonymous antigen vaccines consist of anonymous antigen vaccines ex vivo and anonymous antigen vaccines in vivo, which utilize all the tumor antigen profiles in a patient to act as a vaccine.

Tumor vaccine platforms can be classified into four types based on their preparation methods and sources of tumor antigen: cellular vaccines, viral vaccines, peptide vaccines, and nucleic acid vaccines [12,13]. Cell-based vaccines are the main form of original tumor vaccines, with DC vaccines showing significant results in clinical trials [14]. Viral vaccines utilize viruses as vectors to treat and prevent tumors. Peptide vaccines consist of known or predicted tumor antigen epitopes and typically exhibit lower immunogenicity, thus are combined with adjuvants to enhance their immunogenicity [15,16]. Nucleic acid vaccines include DNA and RNA vaccines composed of genes encoding pathogen antigens and vector groups [17]. DNA vaccines are closed circular DNA plasmids encoding tumor-associated antigens (TAAs) or immunomodulatory molecules to induce tumor-specific responses; mRNA vaccines are synthesized in vitro and can encode antigens post-internalization to stimulate immune responses [18]. Based on our understanding of tumor-specific immunogenic antigens, the number of patients expressing corresponding tumor-specific antigens, and how these antigens co-localize with professional APCs, tumor vaccines can further be divided into predefined (known) antigen vaccines and anonymous (unknown) antigen vaccines. Predefined antigen vaccines are categorized into shared antigen vaccines—expressed in multiple patients’ tumors—and personalized antigen vaccines tailored for individual patients. Shared antigen vaccines are prepared with antigens widely expressing in a group of patients, thus may not be effective for some patients lacking the targeted antigen. Differently, personalized antigen vaccines are prepared with antigens derived from a certain patient and therefore could be more specific and effective, which also requires higher costs. Anonymous antigen vaccines can be classified as anonymous antigen vaccines ex vivo or anonymous antigen vaccines in situ that co-localize with APCs either in vitro or at the tumor site [19] (Figure 1). Anonymous antigen vaccines ex vivo refer to the transfusion of autologous APCs which are stimulated by lysates of excised tumor tissues or cells ex vivo, while anonymous antigen vaccines in situ refer to vaccines that promote the release of tumor antigens in situ and activate endogenous APCs.

## 2. Predefined Antigen Vaccines and Challenges of Their Therapeutic Applications

### 2.1. Predefined Antigen Vaccines

In cancer immunotherapy, predefined antigen vaccines have shown promising results. They can be classified into shared and personalized antigens based on their expression across patient populations [19]. Shared antigens are those expressed in a certain proportion of patients, for which corresponding standard tests are utilized [20]. For example, the novel epitope TSA epidermal growth factor receptor variant III (EGFRvIII) is expressed in approximately 25% of glioblastomas [21]. Another notable example are the human papillomavirus (HPV) E6 and E7 proteins, which are found in about 60% of oropharyngeal cancers and nearly all cervical cancers [22]. These shared antigens can serve as targets for therapeutic vaccines that are applicable to multiple patients with similar tumor characteristics. In contrast, personalized neoantigens are unique to each patient and require custom preparation. A notable example is autogene cevumeran, an mRNA-based personalized cancer vaccine developed by BioNTech (Mainz, Germany) that targets up to 20 unique neoantigens identified from each patient’s tumor. Clinical trials have shown that this vaccine can induce durable polyfunctional neoantigen-specific effector CD8^+^ T-cell responses against pancreatic cancer. This personalized approach could elicit stronger antitumor immune responses while minimizing the risk of off-target effects [23]. Recent initiatives like the NHS’s personalized cancer vaccine program aim to tailor treatments based on individual patient profiles, enhancing specificity and effectiveness. The landscape of therapeutic cancer vaccines is evolving, with ongoing clinical trials examining their efficacy across various malignancies such as lung cancer, breast cancer, and melanoma [24]. Despite their potential, therapeutic cancer vaccines face challenges such as overcoming the immunosuppressive tumor microenvironment (TME) and ensuring robust immune responses against diverse tumor antigens. Continued research is essential for optimizing vaccine design and improving patient outcomes in cancer therapy.

### 2.2. Challenges of Predefined Antigen Vaccines Therapeutic Applications

#### 2.2.1. Tumor Endogenous Drug Resistance

Endogenous drug resistance in tumors refers to the inherent ability of cancer cells to resist the effects of therapeutic agents before any treatment is administered. This type of resistance can arise from various factors, including pre-existing genetic mutations, tumor heterogeneity, and the activation of cellular defense mechanisms [25]. For instance, mutations in genes such as KRAS can lead to the production of neoantigens, which may not be effectively targeted by existing vaccines, thus allowing tumors to evade immune detection [26]. The exhaustion of tumor antigens represents a potential immune evasion mechanism, particularly when the depletion of certain antigens does not affect the survival of tumor cells [27]. Additionally, tumor heterogeneity plays a crucial role in resistance mechanisms. Different subpopulations of cancer cells within a single tumor may express varying levels of TAAs or exhibit different immune responses, complicating the effectiveness of vaccines designed to elicit a uniform immune response [28]. Another significant factor contributing to endogenous drug resistance is the activation of intracellular signaling pathways that enhance cell survival and promote DNA repair mechanisms. For example, the activation of the WNT/β-catenin pathway has been linked to reduced immune cell infiltration and poor responses to immune checkpoint blockade therapies [29].

#### 2.2.2. Tumor Exogenous Drug Resistance

The tumor microenvironment (TME) can contribute to exogenous drug resistance by creating an immunosuppressive milieu that inhibits effective immune responses. The presence of cancer-associated fibroblasts (CAFs) and tumor-associated macrophages (TAMs) can alter the extracellular matrix and secrete various cytokines that enhance tumor cell survival and promote resistance to therapies [30]. In terms of specific examples related to cancer vaccines, research indicates that while therapeutic vaccines can stimulate immune responses against tumors, their effectiveness can be significantly compromised by the immunosuppressive factors present in the TME. For instance, studies on neoantigen vaccines have shown effectiveness in eliciting strong T-cell responses; however, their success is often limited by the presence of regulatory T cells (Tregs) and other immunosuppressive elements within the TME [31]. Factors such as hypoxia, acidity, and the presence of regulatory T cells can suppress T-cell activation and function, diminishing the overall efficacy of a cancer vaccine. For example, studies have shown that tumors often produce immunosuppressive cytokines that can inhibit T-cell proliferation and activity, thereby allowing cancer cells to escape immune surveillance [32]. Moreover, a study discusses how certain neoantigen vaccines can induce immunosuppressive Tr1 cells, which are distinct from traditional Tregs but also inhibit tumor elimination [33]. This highlights the complexity of immune responses elicited by neoantigen vaccines.

#### 2.2.3. Limitations of Predefined Antigen Vaccines and Technical Challenges in Research and Development

Neoantigen vaccines can barely eliminate malignant tumors, partly because some selected neoantigens, for example, melanocyte differentiation antigens, cannot induce continuous and effective tumor regression [34]. When the target neoantigen is a mutated subclone of a gene, a tumor vaccine can only kill a few of the tumor cells, which restricts its clinical efficacy. Personalized neoantigens can elicit tumor-specific T-cell responses; however, once the tumor cells expressing these specific neoantigens are eliminated, tumor cells that do not express the neoantigens would continue their expansion [12]. Though some results of neoantigen vaccination have brought hope to cancer treatment, experiments detecting tumor-specific cells in tumor and peripheral blood T-cell populations indicate that only a small fraction of the predicted useful neoantigens possess spontaneous and durable immunogenicity [35,36]. Therefore, our understanding of epitopes that elicit immunogenic responses remains inadequate, and there are still many obstacles in the selection of neoantigens.

Firstly, the types and quantities of neoantigens observed in each tumor sample are currently limited by sequencing and bioinformatics technologies. Traditional methods may miss more than half of the tumor neoantigens due to potential defects in tumor DNA repair and RNA splicing [37]. Secondly, determining which neoantigens can be presented on MHC molecules and whether they possess immunogenicity remains an unresolved issue. In trials involving multiple neoantigen-targeting vaccines, only 15–30% of the predicted epitopes were able to generate CD8^+^ T-cell responses, and these responses were relatively weak [38]. Moreover, some tumor vaccines constructed from antigens with low MHC I binding scores have been observed to induce effective antitumor effects, highlighting the shortcomings of current algorithms [39]. Consequently, therapeutic cancer vaccines aimed at treating existing diseases face challenges that are starkly different from those encountered by preventive vaccines targeting infectious pathogens [40].

## 3. Anonymous Antigen Vaccines

### 3.1. Anonymous Antigen Vaccines Ex Vivo

The development of anonymous antigen vaccine ex vivo starts from isolating the dendritic cells (DCs) of patients, which are then stimulated with specific antigens in vitro (ex vivo) before being reinfused into the patient. The use of autologous DCs allows for a personalized approach to vaccination, as the therapy is tailored to the individual’s unique tumor antigen profile [41,42,43]. This customization aims to enhance immunogenicity and therapeutic efficacy. By capturing a wide range of tumor antigens, anonymous antigen vaccines ex vivo can potentially elicit a more comprehensive immune response compared to vaccines targeting predefined antigens [44]. This characteristic is particularly advantageous in addressing tumors. Kandalaft et al. [41] conducted a Phase I clinical trial evaluating a dendritic cell vaccine pulsed with autologous oxidized lysate in patients with recurrent ovarian cancer, in which their results demonstrated that this approach was well-tolerated and capable for inducing specific immune responses, with a subset of patients exhibiting prolonged progression-free survival. This study provides foundational evidence for the safety and immunogenicity of dendritic cell-based therapies in ovarian cancer. Chiang et al. [44] further advanced this field by reporting a dendritic cell vaccine pulsed with hypochlorous acid-oxidized ovarian cancer lysate. Their research revealed that this vaccine could elicit broad antitumor immunity in preclinical models and early clinical trials, effectively priming T-cell responses against multiple tumor-associated antigens. This work highlights the importance of optimizing antigen presentation to enhance the efficacy of dendritic cell vaccines. Dhandapani et al. [43] explored the synergistic effects of autologous cervical tumor lysate-pulsed dendritic cell stimulation combined with cisplatin treatment. Their results indicated that this combination therapy could significantly reduce the population of regulatory T cells in vitro, thereby enhancing antitumor immunity and suggesting a potential strategy for overcoming immune suppression in the tumor microenvironment. Nakazawa et al. [42] investigated the capacity of human dendritic cells pulsed with autologous induced pluripotent stem cell (iPSC) lysate to induce cytotoxic T lymphocytes (CTLs) against HLA-A33-matched cancer cells. This study demonstrated effective CTL activation, underscoring the potential for utilizing iPSC-derived antigens to personalize immunotherapeutic strategies. These studies highlight the potential efficacy of dendritic cell vaccines in enhancing immune responses against tumors. Overall, compared to predefined antigen vaccines, anonymous antigen vaccines ex vivo could provide a comprehensive spectrum of tumor antigens and demonstrate significant efficacy in inducing systemic tumor regression [45].

### 3.2. Anonymous Antigen Vaccines In Situ

The development of tumor vaccines has been limited due to the lack of universal tumor-associated antigens (TAAs) that could activate tumor-specific TILs against the heterogeneity and different kinds of tumor, along with the challenges associated with isolating and preparing personalized vaccines. Therefore, in situ vaccination methods, which aim to initiate or enhance immune responses at the tumor site by utilizing the abundant potential tumor-associated antigens present locally, have garnered significant interest. This approach is referred to as in situ vaccination (ISV) [46]. There are various types of anonymous antigen vaccines in situ, including viruses, pattern recognition receptor (PRR) agonists, other immune stimulators, oncolytic viruses, and oncolytic peptides [47,48,49,50]. These vaccines must effectively induce the recruitment of antigen-presenting cells (APCs), facilitate tumor antigen loading, and activate these cells to successfully initiate the cross-priming of tumor-reactive T cells, thereby inducing systemic antitumor immune responses or vaccine effects [19]. In situ vaccination utilizes the full spectrum of tumor antigens to elicit comprehensive antitumor immunity. Representative results from clinical trial reports are summarized in Table 1.

#### 3.2.1. Flt3L

Flt3L is a dendritic cell (DC) growth factor that can induce rapid DC expansion in vivo. Signals mediated by Flt3L through its receptor Flt3 are essential for the survival, proliferation, and differentiation of DC progenitors and immature DCs. Flt3L promotes the maturation and activation of DCs, enhances the expression of the co-stimulatory molecules and cytokines that facilitate T-cell activation and differentiation, and promotes the migration of DCs to lymphoid organs and other tissues, thereby presenting antigens to T cells and triggering immune responses. Consequently, Flt3L has been utilized as a tool to increase DC numbers in tumor immunotherapy, potentially enhancing the efficacy of such treatments [49,61]. Clinical trials have validated the antitumor efficacy of Flt3L, in which a phase I clinical study reported that injection of an adenoviral vector expressing herpes simplex virus thymidine kinase (HSV-TK) and Flt3L into the tumor cavity of resectable malignant gliomas induced effective antitumor immune responses, resulting in the generation of antitumor memory and new antigen recognition capabilities. Preliminary results indicate that combination therapy with the viral vector improved survival rates, although further research and analysis are required. This clinical trial has received approval from the FDA and multiple institutions and is currently ongoing (NCT01811992) [62]. Overall, these studies suggest that Flt3L may be an effective tool for in situ tumor vaccination, as it can enhance immune responses to tumor antigens and increase the numbers of DCs and other antigen-presenting cells. However, further research is needed to determine the optimal dosing regimen and timing for Flt3L administration, as well as its efficacy in large-scale clinical trials.

#### 3.2.2. TLR Agonists

Toll-like receptors (TLRs) are a class of evolutionarily conserved pattern recognition receptors (PRRs) that play a crucial role in cancer immunotherapy [63]. TLR ligands possess potent antigen-presenting cell (APC) activation capabilities, thereby promoting the induction of antitumor immune responses. They have been utilized as adjuvants in conventional vaccines to enhance the efficacy of tumor immunotherapy [64].

TLR9 is an endosomal receptor that is highly expressed in various mouse DC subsets and predominantly expressed in human B cells and plasmacytoid DCs, but not in conventional DC1 cells that have antigen cross-presentation capabilities. Most TLR9 agonists are unmethylated, low-methylation CpG oligodeoxynucleotides, which can be classified into the categories CpG-A, CpG-B, or CpG-C. These agonists induce the activation of plasmacytoid DCs and B cells, leading to the production of pro-inflammatory cytokines such as type I interferon (IFN). However, a large phase III trial reported an objective response rate (ORR) of 9% for the combination of CpG-B tilsotolimod with ipilimumab in treating refractory melanoma, which is similar to ipilimumab alone. In a trial involving the injection of virus-like particles containing CpG-A (CMP-001) in patients with anti-PD-1 refractory melanoma, monotherapy led to systemic tumor regression, while the combination with pembrolizumab yielded an ORR of 28%. These trials reflect a common issue that while TLR9 agonists can induce intratumoral inflammatory responses, their efficacy remains suboptimal, potentially due to insufficient presentation of tumor antigens. Tumor antigens are primarily presented by cDC1 to CD8^+^ T cells; however, cDC1 does not express TLR9 and therefore cannot be activated by CpG-A.

TLR3 is primarily expressed on cDC1 and recognizes double-stranded RNA [65]. Poly-ICLC, a complex of polyinosinic-polycytidylic acid, poly-L-lysine, and carboxymethylcellulose, is a widely studied TLR3 agonist that activates different APC subsets through TLR3 and RIG-I-like receptors (RLRs), such as MDA-5. Several peptide-based brain cancer vaccine studies suggest that Poly-ICLC applied in situ may produce positive therapeutic effects [66,67,68]. Additionally, intratumoral injection of Poly-IC significantly inhibited tumor growth and extended survival in mice with melanoma; when combined with tumor-specific T cells or CD40L-expressing plasmids, complete tumor elimination was achieved [69].

The primary ligand for TLR4 is bacterial lipopolysaccharide (LPS), which activates DCs upon binding to TLR4, enhancing antigen presentation and producing various pro-inflammatory cytokines [70,71]. In rodent tumor models, intratumoral injection of LPS has demonstrated antitumor effects without significant toxicity. In subcutaneous glioblastoma mouse models or subcutaneous glioma rat models, intratumoral injection of LPS led to partial and complete tumor regression. Notably, nude mice exhibited lower levels of tumor regression, indicating that T cells play a crucial role in the tumor regression process [72,73]. Another TLR4 agonist (OK-432/Picibanil) used in combination with intratumoral DC injection therapy for pancreatic cancer is currently undergoing clinical trials (NCT00795977). Similarly, a recent study has demonstrated that in situ vaccines can elicit robust immune responses. For instance, the use of cowpea mosaic virus (CPMV) as an in situ vaccine has shown promising results in mouse models, inducing systemic antitumor immunity and triggering regression in both treated and untreated tumors. This strategy also enhances responses to anti-PD-1 therapy, suggesting a synergistic effect when combined with checkpoint inhibitors [74].

#### 3.2.3. Intratumorally Administered Oncolytic Viruses, Oncolytic Bacteria and Oncolytic Peptides

Oncolytic viruses (OVs) is regarded as a promising new class of tumor immunotherapy, which utilize the inherent ability of certain replicative viruses to preferentially infect and lyse tumor cells, demonstrating potential systemic vaccine effects following intratumoral administration [15]. The main advantage of oncolytic viruses is their ability to be engineered to express specific genes, enhancing cross-presentation of tumor antigens, improving the maturation of antigen-presenting cells (APCs), and reducing immune suppression within the tumor microenvironment [46]. Currently, the only oncolytic virus approved by the FDA is Talimogene laherparepvec (TVEC), a genetically modified type 1 herpes simplex virus characterized by reduced virulence and selective replication within tumors. A phase III randomized trial comparing TVEC with GM-CSF demonstrated a significant increase in the objective response rate (ORR) for TVEC [47]. Additionally, a multicenter phase II study indicated that TVEC could induce the regression of non-injected lesions [75]. Despite many strategies showing antitumor efficacy in mouse models and various cancer types, response rates remain highly variable [46].

Similarly to oncolytic viruses, research has explored the use of oncolytic bacteria for tumor immunotherapy. For instance, researchers developed a photosensitizer-labeled anaerobic oncolytic bacterium based on metabolic engineering. After injection into melanoma tumors, these anaerobic bacteria proliferate in hypoxic regions of the tumor, competing for nutrients and thereby limiting tumor growth and causing the lysis of the tumor cells. The photosensitizer on the bacteria exerts photodynamic effects in oxygen-rich areas to further eliminate residual melanoma. This engineered oncolytic bacterium effectively eradicated melanoma in mice and exhibited ideal biocompatibility, presenting a promising approach for eradicating solid tumors [76].

Furthermore, increasing evidence supports the value of oncolytic peptides as anticancer agents. Despite intra-tumor heterogeneity (ITH), oncolytic peptides can induce robust antitumor immune responses by promoting the infiltration of cytotoxic T lymphocytes (CTLs) and other immune effector cells into tumors while preferentially killing malignant cells [50]. Some oncolytic peptides can trigger immunogenic cell death (ICD), activating and recruiting immune effector cells [77]. In a phase I dose-escalation study involving intratumoral administration in patients with advanced solid tumors, the oncolytic peptide LTX-315 demonstrated ideal patient tolerance and therapeutic efficacy, including tumor regression in some patients and a significant reduction in tumor volume in 30% of cases, promoting CD8^+^ T-cell infiltration within tumors and inducing distant responses [59]. However, clinical development of these agents for tumor treatment remains in its early stages [50].

## 4. Advantages of Anonymous Antigen Vaccines In Situ

In situ vaccination (ISV) represents a method of forming tumor vaccines in vivo, which significantly reduces the technical obstacles in ex vivo vaccine development, thereby facilitating faster vaccine development. Furthermore, this strategy is not limited to single tumor-associated antigens (TAAs) or tumor-specific antigens (TSAs), but rather can utilize all the tumor antigens in a patient. To elicit a robust memory antitumor immune response, an in situ vaccine should be capable of inducing immunogenic cell death (ICD) in tumors, promoting the release of TAAs and enhancing antigen-presenting cell (APC) uptake and activation, which in turn induces T-cell responses and activates systemic antitumor immunity. Since fully activated effector T cells can kill tumor cells independently of co-stimulatory signals and are less sensitive to inhibitory signals, potent antitumor T cells generated at one tumor site can also attack distant tumor lesions. Therefore, an effective in situ vaccination strategy requires the treatment of a single tumor site to induce the release of tumor antigens and the occurrence of ICD, leading to a systemic immune response and tumor regression, with this delivery site being personalized for each patient [40]. As immunotherapy increasingly becomes a core component of cancer treatment, there is a growing demand for methods to enhance the responsiveness to immune checkpoint blockade (ICB) therapies [40]. Anonymous antigen vaccines in situ are likely to be the most promising approaches to achieve this goal by transforming patients from low-T-cell infiltration to a high-T-cell infiltration phenotype, thereby improving the responsiveness to ICB therapies [78,79].

## 5. Conclusions

The primary objective of cancer immunotherapy is to induce a strong antitumor immune response without affecting normal cells in the body. The encouraging efficacy demonstrated by predefined antigen vaccines has opened new avenues for cancer immunotherapy; however, techniques to predict, identify, and screen for the correct antigen epitopes remain insufficient. Anonymous antigen vaccines in situ can leverage the complete tumor antigen repertoire from cancer patients, providing a clearer individual tumor profile that current technologies could not offer while minimizing the risk of immune escape due to tumor variability. The next generation of in situ vaccines has shown considerable efficacy as a new direction in tumor vaccine therapy, and further evaluation of the immune effects and clinical efficacy induced by various in situ vaccination strategies across different tumor types is still needed.

## Figures and Tables

**Table 1:** Anonymous Antigen Vaccines In Situ Entering Clinical Trials.

Platform	Cancer	Trial Phase	Overall Number of Participants	Outcome	References
Flt3L	GBM	I	18	The 12-month overall survival was 72.2% and the 24-month overall survival was 38.8%	[51]
	Lymphoma	II	11	8/11 patients with tumor regressions, 3/11 with abscopal response	[52]
Bempegaldesleukin	Cell Renal Cell Carcinoma	III	311	ORR was 23.0%	[53]
TLR9 agonist	Melanoma	III	481	1/243 patients with CR, 20/243 patients with PR, 45/243 patients with SD in Arm A: Ipilimumab, 1/238 patients with CR, 20/238 patients with PR, 64/238 patients with SD in Arm B: IMO-2125 Plus Ipilimumab	[54]
TLR3 agonist	Solid Tumors	I	13	3/13 patients with PR, 10/13 with SD	[48]
	Pancreatic Cancer	I	27	Median PFS was 13 months with rintatolimod, versus 8.6 months in the matched control. The median OS was 19 months with rintatolimod, versus 12.5 months in the matched control	[55]
TLR4 agonist	Pancreatic Cancer	I	9	5-year survival rate of 2/9, without recurrence of the disease	[56]
	Metastatic soft tissue sarcomas	I	12	1/12 patients with CR	[57]
TVEC	Melanoma	III		50/295 patients in the TVEC group achieved CR, compared to 1/141 in the GM-CSF group	[47]
Nadofaragene firadenovec	Bladder Cancer	III	103	55/103 patients with CR	[58]
Oncolytic Peptide LTX-315	Solid Tumors	I	27	12/27 patients achieved SD	[59]
VCN-01	Pancreatic Can-cer	I	12	6/12 patients with PR	[60]

GBM, Glioblastoma; ORR: objective response rate; CR, complete response; PR, partial response; SD, stable disease; PD, progressive disease; PFS, progression-free survival; OS, overall survival.

## Data Availability

All data relevant to the study are included in the article. Additional data are available upon reasonable request to the corresponding author.
